# The Influence of Religious Commitment on Social Cognition: An Exploratory Study of Social Representations of Peace Among a Sample of Graduates Cameroonians

**DOI:** 10.5964/ejop.13155

**Published:** 2026-02-27

**Authors:** Jean-Claude Etoundi, Boris Goujon, Sandrine Gaymard

**Affiliations:** 1 Unité de recherche Culture, Éthique, Religion et Société (Céres), Catholic University of Toulouse, Toulouse, France; 2 Laboratoire de Psychologie des Pays de la Loire (LPPL UR 4638), University of Angers, Angers, France; 3 Laboratoire Angevin de Recherche en Ingénierie des Systèmes (LARIS UR 7315), University of Angers, Angers, France; University of Studies of Bari, Bari, Italy

**Keywords:** social representations, peace, Cameroon, religious commitment, structural approach of social representations

## Abstract

The preservation of peace is a key concern of the Cameroonian people and the subject of debate within the political class. The public’s marked interest in this issue prompted us to look at social representations of peace in Cameroon through the prism of religious commitment. Adopting a structural approach to social representations, we conducted a study among respondents with different levels of religious commitment (*N* = 156). Data collected via free association and characterization questionnaires were submitted to hierarchical and Q-sort analyses. Results indicated that *unity* is the factor that best characterizes peace. A comparative analysis of the organization of representations between groups with different levels of religious commitment failed to reveal any major differences. Based on the elements identified by participants, we argue that *proximity to the object* is the main factor underlying the construction of social representations of peace in Cameroon.

Cameroon’s motto is “Peace, Work, Fatherland”, and maintaining or preserving peace seems to be a key concern for both the government and the Cameroonian people. The omnipresence of this term in the Head of State’s addresses to the nation attests to its importance in Cameroonian society (Ambomo, 2013). This emphasis on the imperative of preserving peace has led some to say that the Cameroonian president is a “man of peace” and the “guarantor of national unity” (Sakho, 2012). However, reactions to these recurring references are far from unanimous. Many people consider that peace in Cameroon is the “peace of the graveyard” (Tsimi Essono, 2012). Others see it as reflecting the population’s resignation in the face of a complex social environment (Soudan, 2012).

Despite differences of opinion, occasional intercommunal tensions, and the emergence of violent secessionist groups in the English-speaking regions, the country remains one of the most stable in the Central African subregion (International Crisis Group, 2019). Cameroon is home to a mosaic of peoples living together in relative harmony. Given that it has been subject to the same constraints that have often plunged other African countries into serious social crises (Bedzigui, 2008), how can we explain this social harmony?

To address this question, we examined social representations (SRs) of peace, as they highlight functions of knowledge, identity, justification, and orientation (Abric, 1976). In other words, they help us understand and explain reality, preserve the specificity of social groups, anticipate and produce expectations, and justify behavior. This approach is particularly relevant given that, to our knowledge, peace in Cameroon has never been analyzed as a social object from a psychosocial perspective, and more specifically through the prism of SRs.

## Theoretical Aspects and Research Problem

In 1961, Moscovici put forward his theory of SRs (SRT), based on Durkheim’s concept of collective representation (Durkheim, 1898; Moscovici, 1961). This theory was intended to account for the processes and contents by which social groups represent reality. In other words, Moscovici considered them to be a collective elaboration of knowledge about objects of collective interest. SRs are therefore one of the modalities of social thinking (Rouquette, 1973).

SRs are a “form of knowledge that is socially elaborated and shared. They have a practical aim and contribute to the construction of a reality common to a social whole” (Jodelet, 1989, p. 36, our translation). They thus provide a framework for analyzing human interactions, enabling us to understand the behavior of individuals and groups based on how they project themselves and their social environment. This involves considering both individual and group perspectives in order to identify the foundations of social thinking and the construction of social relations.

According to the structural approach, SRs are structured around a central core (CC) and a peripheral system (Abric, 2001). The elements making up SRs are organized according to their nature. CC elements are indispensable and non-negotiable, as they ensure the meaning, organization, and stability of the representation, and are generally the subject of consensus. Peripheral elements depend on the core and ensure the coherence of the representation across multiple contexts. They are flexible, reflect individual variability, and are typically the first to evolve when representations change. Flament (1989) suggested that the peripheral system allows different contexts to be decoded, while maintaining a coherent representation.

## Is Peace an Object of SRs in Cameroon?

Research on social representations of peace conducted primarily in Western contexts shows that these are organized around intra-personal and relational considerations, positive emotions, freedoms, and well-being (Etoundi & Gaymard, 2021; Sarrica, 2007; Sarrica & Wachelke, 2010; Van der Linden & Licata, 2012). Given current differences of opinion about the basis of social cohesion in Cameroon, and considering that the objects of SRs are often sources of divergence and debate about the very idea of the social group (Flament & Rouquette, 2003; Moliner, 2011), it is reasonable to assume that issues related to peace are highly salient in Cameroonian society. Moreover, the country’s marked social and cultural diversity complicates any attempt to apprehend social thinking about peace, as it mobilizes multiple layers of identity (Bruneau et al., 2001). Although this diversity has periodically strained social harmony, it has not led, until recently, to large-scale social breakdown. The reasons remain difficult to establish and have not yet been demonstrated conclusively. Nonetheless, everyday life reveals domains in which the population converges; one such domain is religion. Despite the secular character of the State, religion occupies a prominent place in people’s lives. According to the most recent population census, 69.2% of inhabitants are Christians, 20.9% Muslims, 5.6% animists, 1.0% other faiths and 3.2% with no declared religion (United States Department of State, 2023). This religious grounding permeates daily interactions, making religious considerations omnipresent (Etoundi & Gaymard, 2017; Gaymard et al., 2015).

Because religious antagonisms are frequently implicated in intergroup conflict worldwide (Ferguson & McKeown, 2016; Neuberg et al., 2014; Saroglou, 2016), and in Africa, as illustrated by conflicts in the Central African Republic between Christian and Muslim armed groups and even civilian populations (Fancello, 2020), it is important to understand how individuals’ religious commitment shapes cognitions about peace. The present study therefore adopts the framework of social representations theory (SRT) to examine how shared values and symbolic resources contribute to constructing a group’s social identity in relation to peace.

## Study Objective

Research on intergroup relations emphasizes the potentially conflictual nature of such relations, given competition over scarce resources and the tendency toward in-group favoritism (Böhm et al., 2020; Hewstone et al., 2014; Neuberg et al., 2014). Since social representations constitute socially shared knowledge that guides interpretation and prescribes behavior, analyzing social representations of peace can illuminate why Cameroon has, to date, maintained relative stability. Given the centrality of religion in Cameroon, the present study aims to assess the impact of religious commitment on the structure of peace-related SRs. Because social representations influence social practices (Michel-Guillou, 2006; Zbinden et al., 2011), identifying how religious involvement shapes the organization of these representations can help explain attitudes and behaviors relevant to the preservation of social harmony.

## Method

### Participants

We used convenience sampling, ensuring that participants were drawn from the main religious groups in the country. Due to reluctance among many of those initially approached, the final sample comprised 156 participants who freely agreed to take part. The sample included men (*n* = 91) and women (*n* = 65), with half aged between 18 and 30. Regarding employment status, 57.7% reported having a stable job, while 42.3% were either in training, unemployed, or in precarious employment. Participants represented Cameroon’s four cultural areas in the following proportions: 48.07% Fang-Béti, 26.92% Grassfields, 11.53% Coastal, and 13.46% Sudano-Sahelian. Participants were also drawn from the country’s three major religious traditions. They were categorized either as members of classical religions (Catholic, Protestant, Muslim) or of emergent religious denominations, mainly Evangelical churches (Mvessomba, 2008). In this study, we focused on the influence of religious commitment rather than the effectiveness of faith. Religious commitment was operationalized as the frequency of participation in religious rites, which we considered a more objective indicator than subjective adherence to dogma. To assess religious involvement, we developed a three-point scale derived from two items. Participants who identified as religious were first asked whether they practiced the rites of their religion, and then to report the number of days per week they engaged in these practices (0–7). Responses were used to classify participants into three categories:

- Not engaged (no reported practice).
- Occasional (practice fewer than four days per week).
- Regular (practice four or more days per week).

This categorization was designed to reduce potential bias related to social desirability.

On this basis, we identified three groups: engaged believers (regular practice; *n* = 92), moderate believers (occasional practice; *n* = 50), and non-engaged believers (no practice; *n* = 14).

### Material and Procedure

Research on SRs employs diverse methodological approaches. Given the complexity of the present topic, we adopted a multi-method design, also known as methodological triangulation or mixed method (Caillaud & Flick, 2016; Gaymard et al., 2013; Vergès, 2001). This does not simply involve the use of different survey techniques but includes tackling the subject of SR from different angles.

Two tools were used: a free-association-test and a characterization questionnaire (Abric, 2003; Vergès, 2001). In addition, participants provided socio-demographic data. Depending on their language preference (French or English), they completed the questionnaires either individually or face-to-face with the investigator.

For the free associations, participants were asked to spontaneously produce three to five words or expressions in response to the inducer “Peace in Cameroon.” Their responses were read back to ensure accuracy. Following Abric’s procedure, participants then ranked their words in descending order of importance, since the first words mentioned are not always the most salient. This ranking allowed us to situate each element within the SR structure.

Associations were recorded in an Excel.csv file. As recommended by Bouhon (2009) and Ferriere (2009), lemmatization and synonym grouping were applied to reduce lexical variability (e.g., “*love of country*” and “*attachment to the nation*” were coded as *patriotism*; “*living together*” as unity; “*many tribes*” and “*different cultures*” as *cultural diversity*). The resulting corpus was subjected to lexicometric analysis with EVOC 2005 software, which generated SR structures in a four-box table. Following the frequency-rank method, the central core appeared in the upper-left box, the first periphery in the upper-right, contrasting elements in the lower-left, and the second periphery in the lower-right (Vergès et al., 2005).

The characterization questionnaire was based on items derived from a preliminary online free-association study (*N* = 32). From this, we retained the 12 most frequently cited items. The questionnaire required participants to choose the four most characteristic items, followed by the four least characteristic items, from the remaining pool. This forced-choice procedure, grounded in an equiprobability model, allowed us to test the centrality of elements identified in the preliminary study (Vergès, 2001). Responses were transcoded into three values:

- 1 (Least characteristic).
- 2 (Neutral).
- 3 (Most characteristic).

This produced characteristic distribution curves, which can differentiate central, peripheral, and contrasted elements (Gaymard, 2003). Specifically, J-shaped curves indicate central elements, bell-shaped curves indicate peripheral items, U-shaped curves denote contrasted items, and inverted J-shaped curves indicate non-characteristic items.

To avoid priming effects, the free-association task was always administered before the characterization questionnaire. Data from this study are available online at Etoundi et al. (2024).

## Results

The corpus of free associations for the whole sample consisted of 726 words and expressions (i.e., average of 4.65 words per participant including 171 distinct items and 83 hapaxes (11.4% of the corpus). Analysis of the variability (8%) and diversity (31.66%) indices indicated informational redundancy. Given that communication is an important factor in the “construction of a common reality” (Jodelet, 1989, p. 53, our translation), this redundancy and recurrence of stereotyped evocations reflected a form of shared knowledge among participants (Kalampalikis & Moscovici, 2005; Kmiec & Roland-Lévy, 2014). The EVOC analysis, at an intermediate frequency threshold of 20 occurrences and a mean rank of 2.5, showed that the central core contained only the term *unity* (see Table 1). The first periphery included expressions such as *security*, *cultural diversity*, and *social cohesion*. Contrasting elements included *education* and *hospitality*, while the second periphery comprised items such as *governance* and *demagogy*.

**Table 1:** Structure of the Social Representation of Peace of the Whole Sample with the Evoc Software

Mean rank ≤ 2.5				Mean rank > 2.5		
Frequency ≥ 20	Unity **(CC)**	34^a^	2.29^b^	Love	22	3.31
				Wellbeing	30	3.50
				Social cohesion	40	2.87
				Democracy	26	3.07
				Development	36	3.33
				Cultural diversity	25	3.42
				Employment	18	3.03
				Justice	21	2.59
				Individual rights	49	2.85
				Patriotism	29	3.41
				Security	96	2.62
				Solidarity	32	3.15
				Stability **(**1^st^ periphery)	52	2.82
	Education	11	2.45	Governance	18	2.72
**Frequency < 20**	Hospitality (Contrasting items)	12	2.08	Demagogy (2^nd^ periphery)	19	3.15

*Note*. ^a^Frequency ^b^Rank.

**Figure 1:**
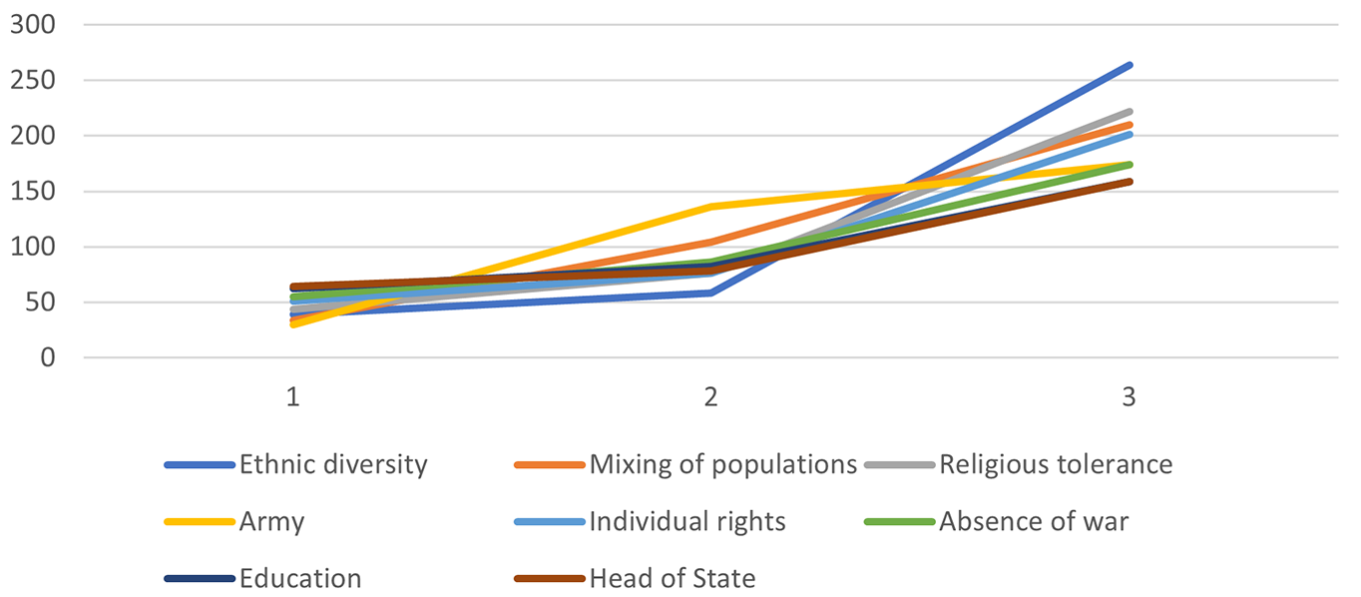
Core Elements of SR of Peace of the Whole Sample, Based on Item Scores on the Characterization Questionnaire

**Figure 2:**
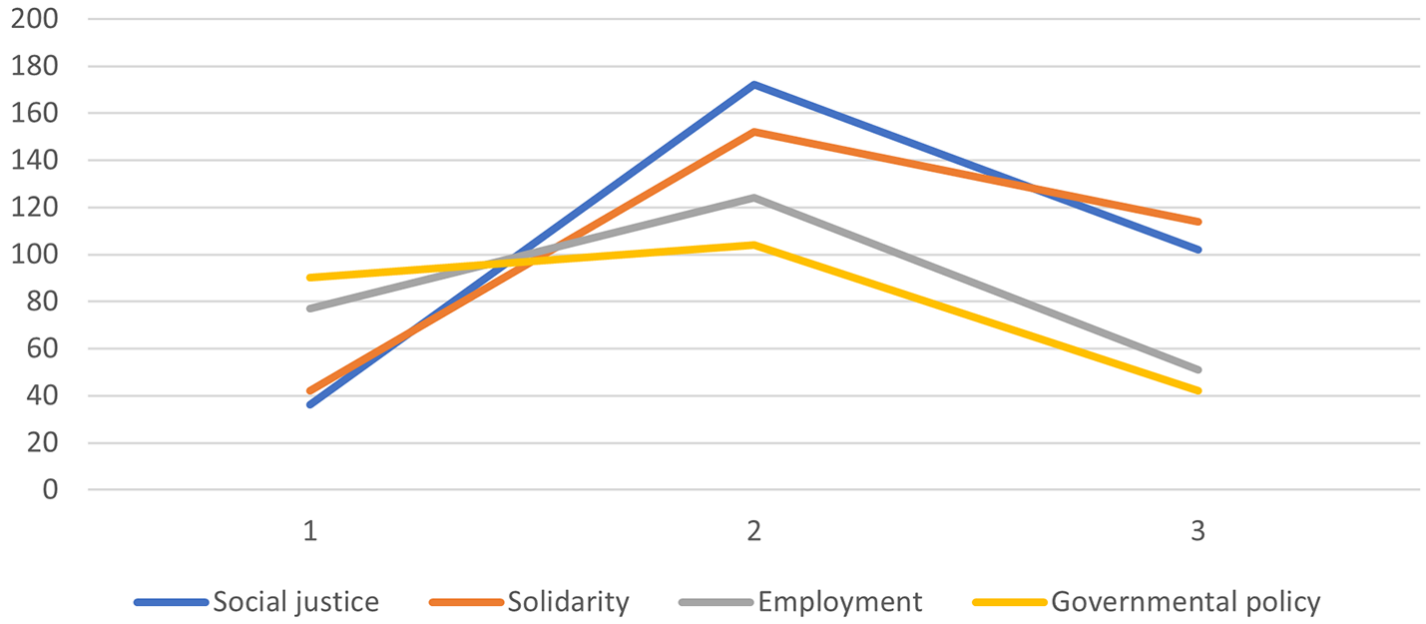
Peripheral Elements of the SR of Peace of the Whole Sample, Based on Item Scores on the Characterization Questionnaire

Responses to the characterization questionnaire further highlighted *ethnic diversity*, *mixing of populations*, *religious tolerance*, *the army*, *freedom of expression*, and *absence of war* as the most characteristic elements of peace. The graphical representation (see Figure 1) showed J-shaped curves for these items, confirming their centrality. In contrast, items such as *employment*, *solidarity*, *social justice*, and *government policy* formed bell-shaped curves (see Figure 2), consistent with their status as peripheral elements.

Taken together, results from both methods converged on the idea that SRs of peace in Cameroon are primarily structured around harmonious coexistence.

Analysis by groups of believers revealed that the diversity of expressions varied with the level of religious commitment (see Table 2, Table 3, Table 4 and Table 5). Engaged and moderate believers produced associations with relatively low diversity, whereas non-engaged believers produced the highest proportion of hapaxes. When analyzed separately with EVOC, each group exhibited a distinct SR structure. For each group, the intermediate frequency and mean rank were defined based on significant differences observed across groups (Gaymard & Bordarie, 2015).

**Table 2:** Statistical Data of the Corpus of the Groups of Believers

Groups	Number of free associations	Ratio free associations	Number Hapax	Rarity indices	Diversity indices
Engaged believers	444	4.82	16	29.62	12.16
Moderate believers	277	4.78	13	31.70	17.15
Non-engaged believers	47	3.33	4	30.76	31.14

**Table 3:** Structure of SR of Peace of Engaged Believers

Mean Rank ≤ 2,5				Mean Rank > 2,5		
Frequency ≥ 13	Stability	24	2.208	Love	15	3.47
	Unity	21	2.143	Well-being	24	3.50
				Social cohesion	20	3.241
				Democracy	17	2.941
				Development	30	3.200
				Individual rights	36	2.944
				Patriotism	16	3.375
				Security	52	2.692
				Solidarity	14	3.318
				Cultural diversity	14	3.111
Frequency < 13	Employment	11	2.364	Governance	9	3.000
	Hospitality	9	1.889			
	Justice	8	2.250			

**Table 4:** Structure of SR of Peace for Moderate Believers

Mean rank ≤ 2.9				Mean rank > 2.9		
Frequency ≥ 12	Social cohesion	18	2.83	Stability	21	3.24
	Individual rights	20	2.55			
	Security	41	2.56			
Frequency < 12	Governance	8	2.50	Cultural diversity	7	3.25
	Justice	11	2.73	Patriotism	9	3.33
	Solidarity	8	2.34	Tolerance	7	3.86

**Table 5:** Structure of SR of Peace for Non-Engaged Believers

Mean rank ≤ 3.5				Mean rank > 3.5		
Frequency ≥ 6	Unity	6	2.66			
Frequency < 6	Patriotism	4	3.00	Employment	1	4.00
	Democracy	5	3.00	Social cohesion	2	3.50
	Security	3	3.33	Individual rights	3	3.66
	Solidarity	2	2.16			

Analysis of free-association responses given by *engaged* or *non-engaged believers* showed that whereas the CC of each group contained the item *unity*, references to *stability* were specific to the *engaged believers.* By contrast, the *moderate believers* had a CC built around the terms *individual rights*, *security*, and *social cohesion*. The peripheries of the three SRs revealed little overlap, as items outside the core shifted position across groups. A chi-square test on items shared by all three groups showed significant differences for *security* (*p* = .0001), *development* (*p* = .002), and *well-being* (*p* = .029).

**Figure 3:**
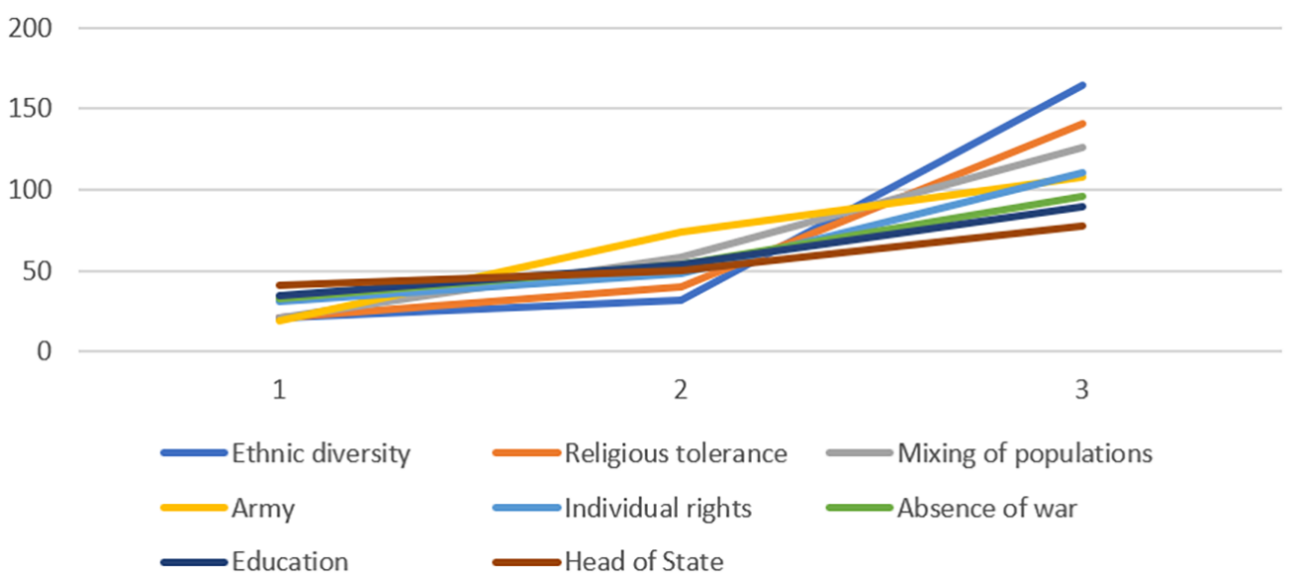
Central Core Elements of SR of Peace of Engaged Believers Based on Item Scores on the Characterization Questionnaire

**Figure 4:**
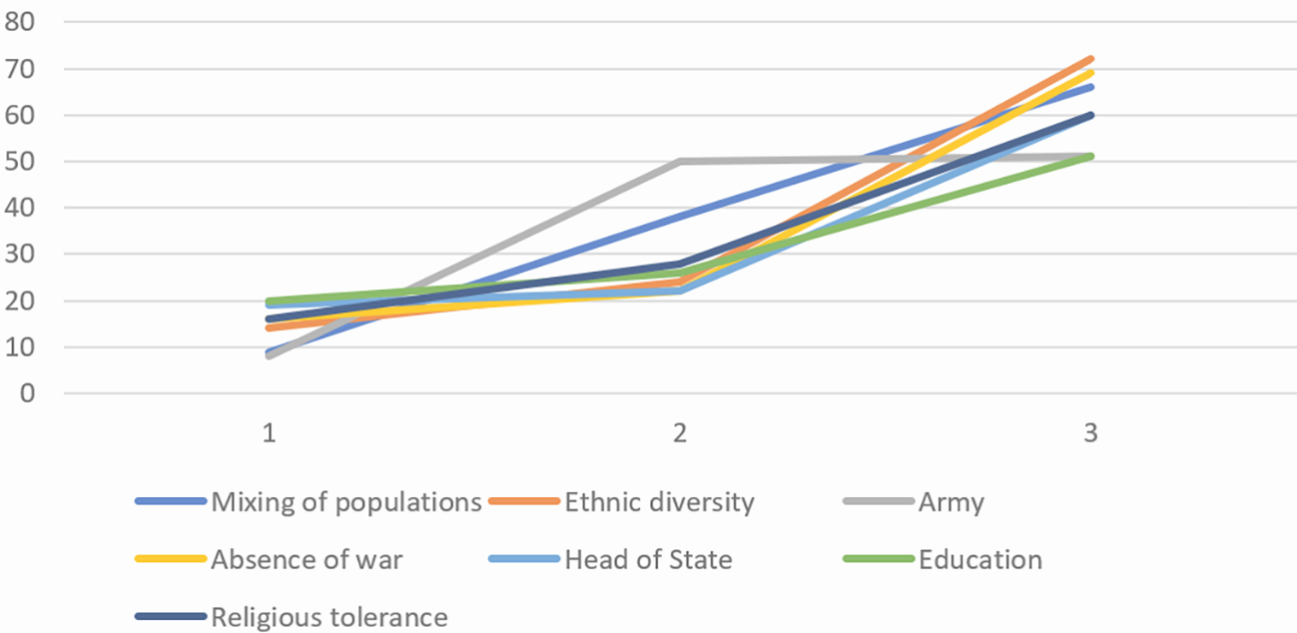
Central Core Elements of SR of Peace of Moderate Believers Based on Item Scores on the Characterization Questionnaire

**Figure 5:**
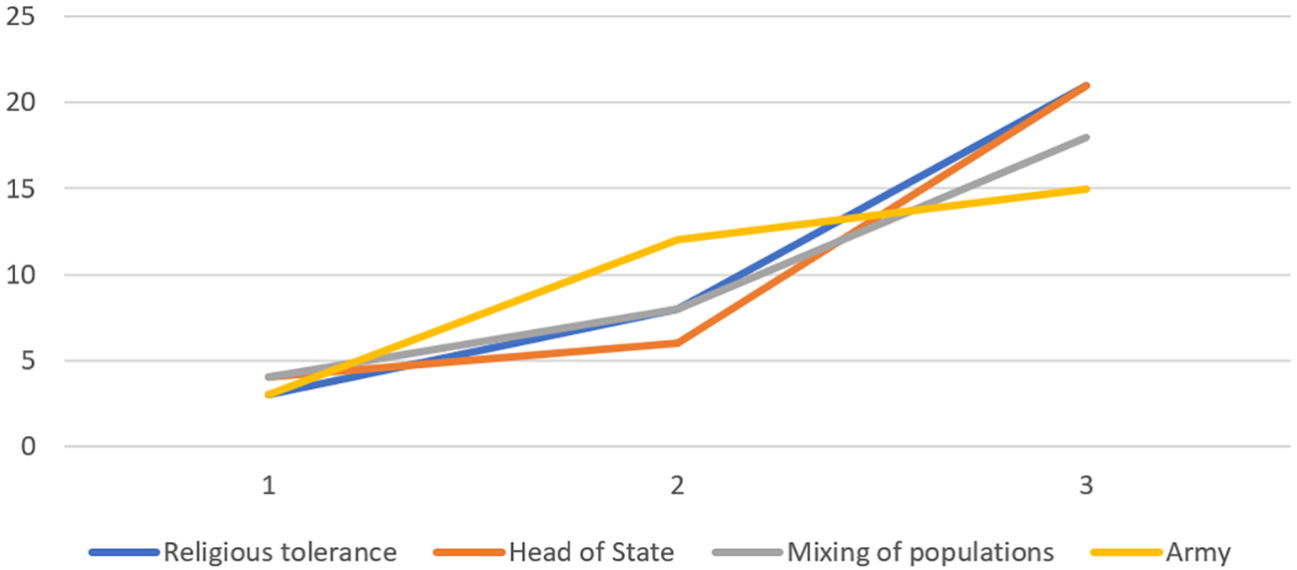
Central Core Elements of SR of Peace of Non-Engaged Believers Based on Item Scores on the Characterization Questionnaire

In the characterization questionnaire (see Figure 3, Figure 4 and Figure 5), items such as *religious tolerance*, *head of state*, *mixing of populations*, and *army* were judged central by all groups. *Ethnic diversity*, *education*, and *absence of war* were identified as central only among engaged and moderate believers. Peripheral elements common across groups included *solidarity*, *social justice*, and *employment*. Moderate believers differed in viewing *individual rights* as a contrasted element, and in excluding *government policy* from their SR.

A one-factor ANOVA on the characterization questionnaire data found no significant relationship between the degree of religious involvement and the importance attributed to individual items.

## Discussion

Given that stability has been a constant feature of Cameroon for decades, despite differences of opinion regarding its extent, we explored social thinking about peace through the lens of social representations (SRs). Two structural tools (free-association test and characterization questionnaire) were used to examine the organization of peace-related SRs according to levels of religious commitment.

Across the whole sample, analysis of free associations showed that unity was the primary element around which the SR of peace was organized. This finding was corroborated by the characterization questionnaire, which highlighted *mixing of populations*, *ethnic diversity*, *religious tolerance*, *the army*, and *the head of state* as the most characteristic items. These elements reflect institutions or symbolic resources that sustain the consensus required for harmonious coexistence among groups. *Education* and *individual rights* also appeared as key factors, acting as catalysts for unity by contributing to individual development and safeguarding democracy as a constitutional right. The centrality of cultural diversity confirmed that “the hybridization of cultures (…) ultimately contributes to the stability of a country where the feeling of nationality includes openness to otherness” (Abada Medjo, 2015, p. 7, our translation). In this respect, SRs of peace in Cameroon can be considered identity markers.

The structure of peace-related SRs that emerged from this study was built around intrapersonal, relational, and emotional factors, similar to findings in Western contexts (Sarrica, 2007; Sarrica & Wachelke, 2010; Van der Linden & Licata, 2012). This suggests that individuals, regardless of cultural setting, may be driven by common ideals concerning the permanence of social stability.

History and context are crucial in the construction of SRs (Beghoura, 2005; Bauer & Gaskell, 1999). Given that Cameroonian independence followed a violent conflict that fractured the social fabric, the salience of *unity* may reflect an implicit agreement among citizens to avoid repeating such traumatic events (Deltombe et al., 2011; Mbembe, 1991; Njeuma, 1989). This interpretation is consistent with the function of SRs in risk management (Gaymard, 2012; Joffe, 2003). Moreover, although Cameroon shares many structural similarities with other African states that have experienced severe crises (Bedzigui, 2008), it has so far avoided widespread instability. This points to a distinctive relationship between the population, its history, and its representations of peace. SRs of peace therefore contribute to the singularization of the Cameroonian population, highlighting the importance of the functions of knowledge, group identification and differentiation of groups relating to this form of social knowledge (Abric, 1976; Breakwell, 2007; Gaymard et al., 2015; Jodelet et al., 1970).

Cross-analyses of SRs by level of religious commitment suggested that peace-related representations were organized around three themes: *unity*, *security*, and *harmonious coexistence*. These themes were common to all three groups of believers. Although the structure of SRs, especially the central core, indicates a different social representation, no fundamental divergences emerged in the general idea assigned by each group. This finding was unexpected, as we anticipated that weak religious commitment might correlate with greater openness to debate, while strong religious commitment would foster homogeneous convictions. Instead, even non-engaged believers produced highly consensual representations. Significant differences observed for *security*, *development*, and *well-being* were largely attributable to disparities in group size. The absence of strong divergence may indicate that belonging to the same national community produces a shared identity that transcends religious practice or commitment (Gaymard, 2003).

The consistent salience of *security*, *unity*, and coexistence across groups underscores their importance as overarching values. These elements resonate with the ethical and moral foundations of religion, rooted in the promotion of good and love among individuals (Bultmann, 1930). Thus, while competition among religious denominations has intensified in recent years, particularly with the growth of evangelical churches (Mayrargue, 2016), this has not translated into divergent representations of peace. Our findings confirm Mvessomba’s (2008) observation that the proliferation of religious groups in Cameroon does not result in significant ideological cleavages.

Some peripheral elements reflected respondents’ broader concerns. Given high unemployment and widespread corruption (Kouagheu, 2023; Mo Ibrahim Foundation, 2023), references to *employment*, *governance*, and *justice* suggest that peace is viewed as contingent on building a fairer social environment. Moreover, whether considering the whole sample or specific groups, the elements identified were closely tied to contemporary socio-economic and political realities. References to *ethnic diversity* and *mixing of populations* reflected the multicultural character of the country, while *security* and the *army* highlighted the role of security forces in the face of threats such as Boko Haram in the north and rebel groups on the border with the Central African Republic. These findings confirm the importance of object proximity in the activation of SRs (Abric, 2001; Abric & Tafani, 1995; Dany, 2016).

### Conclusion

The main finding of this study is that the preservation of unity appears to be the most important characteristic element of the social representation of peace in Cameroon, regardless of participants’ level of religious commitment. Overall, the study showed that peace-related SRs are structured around *unity*, *security*, and *harmonious coexistence*. Despite expectations that religious involvement might generate differences in the organization of these representations, we found no major divergences between engaged, moderate, and non-engaged believers. This convergence suggests that the sense of belonging to a common national entity fosters a shared identity that transcends religious practice and denominational divisions. At the same time, peripheral elements such as *employment*, *governance*, and *justice* reflected pressing socio-economic concerns, indicating that peace is conceived not only as an ideal of coexistence but also as dependent on conditions of fairness, opportunity, and good governance. The results therefore underscore the close relationship between SRs and the contextual realities of participants’ daily lives.

However, several limitations must be acknowledged. First, group sizes were imbalanced, with the non-engaged believers’ group being particularly small, which constrained comparisons. Second, since participants were higher-education graduates, the most accessible population, the findings cannot be generalized to the entire Cameroonian population. Third, because data collection preceded significant socio-political developments, including the rise of secessionist armed groups in Anglophone regions and tensions following the 2018 presidential election, the SRs of peace identified here may have evolved.

Future research should replicate this study with larger, more representative samples and extend it to other African countries with comparable socio-political contexts, to assess whether Cameroon’s configuration is unique. Implementing substitution techniques (Gaymard & Etoundi, 2018), whereby participants infer the representations of other groups, would also allow examination of the extent to which intergroup dynamics shape SRs of peace. Particular attention should be given to contexts marked by community claims and separatist movements, which may reveal divergent perspectives between Anglophone and Francophone populations. Such investigations would help determine whether these events have led to sudden, gradual, or resistant transformations of these representations (Flament, 1989; Flament & Rouquette, 2003; Guimelli, 1989).

In sum, the study highlights that in Cameroon, peace is represented above all as unity in diversity, an identity marker rooted in shared values and historical experience. Religious commitment, while central to daily life and identity, does not fundamentally alter these representations. Rather, it coexists with a broader national consensus, testifying to the resilience of peace as a collective social object.

## Supplementary Materials

**Table:** 

Type of supplementary materials	Availability/Access
Data	
Complete data file.	Etoundi et al. (2024)
Code	
No code was provided.	—
Material	
Items table.	Etoundi et al. (2024)
Data dictionary.	Etoundi et al. (2024)
Study/Analysis preregistration	
The study was not preregistered.	—
Other	
Syntax to recreate data preparation and analyses.	Etoundi et al. (2024)
Caracterization items.	Etoundi et al. (2024)
Curves generation file.	Etoundi et al. (2024)
Free associations whole sample.	Etoundi et al. (2024)

## Data Availability

Data from this study are available online at Etoundi et al. (2024).
